# Supplementary material for: Natural History and Risk Factors of Hymenoptera Venom Allergy in Dogs
Source: Animals (Basel). 2024 Nov 10;14(22):3220. doi: 10.3390/ani14223220 (PMC11591281; doi:10.3390/ani14223220)
Supplement: Supplementary file 1 [file animals-14-03220-s001.zip › animals-3283890-supplementary.pdf]

Table S1. Variables assessed for each dog and all possibilities separated by comma

Category	Variable	Options
**Signalment**	Case number	
	Patient and owner name	
	Age	In years
	Year of presentation	yyyy
	Month of presentation	mm
	Sex	Female, male
	Reproductive status	Castrated, intact
	Breed	Name
	Weight	Kilogram
**History and clinical data**	Age of first sting	In years
	Grade of current sting	0, 1, 2, 3
	Grad of previous sting	0, 1, 2, 3
	Number of total stings	Number
	Interval between stings	mm
	Time of day	Morning, midday, afternoon, evening/night
	Gallbladder wall oedema	Yes, no, did not check
	Alanine Aminotransferase increase	Yes, no, did not check
	Situation	While walking, in garden, on balcony, other,
	Observed insect stings	Yes, suspected, no
**Treatment**	Hospitalization	Yes, no
	Duration of hospitalization	Hours
	Antihistamine	Yes, no, unknown
	Glucocorticoids	Yes, no, unknown
	Gastrointestinal protectants	Yes, no, unknown
	Adrenaline	Yes, no, unknown
	Infusion	Yes, no, unknown
	Venom Immunotherapy	Yes, no
	Other treatments	Analgesics, antiemetics, NSAIDs, antibiotics, dietary supplements, other
**Trigger**	Trigger group	Hymenoptera, food, medication, other
	Specific identification of allergen	Bee, wasp, hornet, bumble, unknown
	Localization of sting	Paw, face, generalized, oral cavity other, unknown
	Provocation	Yes, no
	Allergy testing	Yes, no
	Previous trigger	Bee, wasp, hornet, bumblebee
**Comorbidities**	Atopic dermatitis	Yes, no
	Infectious bowel disease	Yes, no
	Other	Name
	Concurrent medication	Name

Table S2. Questionnaire for owners with dogs stung by Hymenoptera

Questions	Possible answers
Has your dog been stung again by an Hymenoptera?	Yes, no, maybe
By which insect was your dog stung?	Bee, wasp, bumblebee, hornet, unknown
How much time passed between the separate stings?	< 2 months, 2-12 months, 1-3 years, > 3 years, unknown
In which context did the sting occur?	While walking, in the garden, on the balcony, other location
Where was your dog stung?	Paw, head, oral cavity, other
What symptoms did your dog exhibit after the sting?	Itching, swelling, redness of the skin, single episode of diarrhea or vomiting, multiple episodes of diarrhea or vomiting, dyspnea, loss of consciousness, collapse, pallor, no symptoms, other
How did the reaction compare to the previous one?	Milder, same, more severe, unknown
What did you do after re-sting?	Administration of medication, injection of epi-pen, veterinary visit, stinger extraction, other
Have your dog's insect stings affected your quality of life?	Yes, no
Have you changed your lifestyle due to your dog's insect stings?	Yes, no
Has your dog developed new allergies since the first sting?	Yes, food, environmental allergens, medication, other, no
Does your dog like to play with insects?	Yes, no
Do you own an epi-pen for your dog?	Yes, no
Have you heard of allergen-specific immunotherapy?	Yes, no
Do you think an insect allergy can be cured by allergen-specific immunotherapy	Yes, no, I don't know
Image bee	Which insect do you recognize?
Image bumblebee	Which insect do you recognize?
Image wasp	Which insect do you recognize?
Image hornet	Which insect do you recognize?

Table S3. List of affected breeds

Variable	Parameter
**Breed, no. (%)**	
Purebred	143 (80)
Mixed	35 (20)
French bulldog	18 (10)
Dachshund	10 (6)
Labrador retriever	8 (4)
Yorkshire terrier	7 (4)
Maltese	6 (3)
Spitz	5 (3)
Australian shepherd	4 (2)
Boston terrier	4 (2)
Boxer	4 (2)
Chihuahua	4 (2)
Dobermann	4 (2)
Cocker spaniel	4 (2)
German shepherd	4 (2)
Golden retriever	4 (2)
Poodle	4 (2)
Pug	4 (2)
Rhodesian ridgeback	4 (2)
Siberian husky	4 (2)
Chinese crested dog	3 (2)
Continental bulldog	3 (2)
Coton de Tuléar	3 (2)
Miniature pinscher	3 (2)
Parson Russel terrier	3 (2)
Shiba	3 (2)
Barbet	2 (1)
Toypoodle	2 (1)
Australian cobberdog	1 (1)
Australian terrier	1 (1)
Belgian shepherd	1 (1)
Bologneser	1 (1)
English bulldog	1 (1)
English springer spaniel	1 (1)
Entlebucher mountain dog	1 (1)
Finnish lapphund	1 (1)
Flat coated retriever	1 (1)
Havanese dog	1 (1)
Kromfohrländer	1 (1)
Miniature bull terrier	1 (1)
Miniature schnauzer	1 (1)
Prague rattler	1 (1)
Ratonero Bodeguero Andaluz	1 (1)
Rottweiler	1 (1)

Small Münsterländer	1 (1)
Viszla	1 (1)
Welsh terrier	1 (1)

Table S4. Bivariate analysis (fisher's exact test) of factors associated with severe anaphylaxis.

Variables	Number of dogs	Odds ratio	CI 95%	p- value
Age < 2 years	100	1.69	0.87 – 3.33	0.12
Weight < 10 kg	91	1.58	0.82 – 3.06	0.17
Male sex	71	1.14	0.58 – 2.20	0.75
Purebred	143	1.96	0.82 – 5.13	0.12
> 1 episode	43	1.26	0.49 – 3.16	0.66
< 2m interval	12	1.72	0.44 – 6.73	0.37
Previous systemic reaction	10	0.82	0.12 – 5.51	1
Time of the day of the sting	99	1.31	0.65 – 2.68	0.51
Sting in oral cavity	35	3.02	1.32 – 7.08	<0.01*
Bee venom	55	1.79	0.89 – 3.60	0.09
CAD	7	0.27	0.01 – 2.27	0.26
Comorbidity	14	1.73	0.49 – 6.08	0.39
